# Additive Manufacturing of Isotropic NdFeB PPS Bonded Permanent Magnets

**DOI:** 10.3390/ma13153319

**Published:** 2020-07-26

**Authors:** M. Parans Paranthaman, Volkan Yildirim, Tej Nath Lamichhane, Benjamin A. Begley, Brian K. Post, Ahmed A. Hassen, Brian C. Sales, Kinjal Gandha, Ikenna C. Nlebedim

**Affiliations:** 1 Oak Ridge National Laboratory, Oak Ridge, TN 37831, USA; volkanyildirim48@gmail.com (V.Y.); lamichhanetn@ornl.gov (T.N.L.); benjamin.begley@ufl.edu (B.A.B.); postbk@ornl.gov (B.K.P.); hassenaa@ornl.gov (A.A.H.); salesbc@ornl.gov (B.C.S.); 2 Critical Materials Institute, Ames Laboratory, Ames, IA 50011, USA; kgandha@ameslab.gov (K.G.); nlebedim@ameslab.gov (I.C.N.)

**Keywords:** NdFeB PPS bonded permanent magnets, additive manufacturing, thermal stability, tensile strength, magnetic properties

## Abstract

Extrusion based additive manufacturing of polymer composite magnets can increase the solid loading volume fraction with greater mechanical force through the printing nozzle as compared to traditional injection molding process. About 63 vol% of isotropic NdFeB magnet powders were compounded with 37 vol% of polyphenylene sulfide and bonded permanent magnets were fabricated while using Big Area Additive Manufacturing without any degradation in magnetic properties. The polyphenylene sulfide bonded magnets have a tensile stress of 20 MPa, almost double than that of nylon bonded permanent magnets. Additively manufactured and surface-protective-resin coated bonded magnets meet the industrial stability criterion of up to 175 °C with a flux-loss of 2.35% over 1000 h. They also exhibit better corrosion resistance behavior when exposed to acidic (pH = 1.35) solution for 24 h and also annealed at 80 °C over 100 h (at 95% relative humidity) over without coated magnets. Thus, polyphenylene sulfide bonded, additively manufactured, protective resin coated bonded permanent magnets provide better thermal, mechanical, and magnetic properties.

## 1. Introduction

Additive Manufacturing (AM) is a promising technique for bonded permanent magnet (PM) production. It is a novel three-dimensional (3D) materials printing technology in a layer-by-layer fashion. There are several approaches of implementing the AM of the PMs, such as fused deposition modeling (FDM) [1,2,3,4,5,6], selective powder-bed fusion using laser also known as selective laser melting (SLM) [7], spark plasma sintering (SPS) [8], cold spray (CS) [9], stereolithography (SL) [10], direct energy deposition (DED) [11], vat photopolymerization [12], laminated object manufacturing (LOM) [13], and binder jetting technology (BJT) [14]. Extrusion deposition: FDM based AM of bonded PMs have demonstrated useful metrics in terms of energy density, flexural strength, and lower eddy current loss [2,4]. A bonded PM is desired to have a high energy product, good mechanical/flexural strength, high thermal stability, high resistivity, and low eddy current loss. High energy product can be achieved with a higher loading proportion of magnetic fillers in the polymers. The energy product of bonded PMs is proportional to the square of magnetic filler’s volume fraction. Nylon (polyamides) and polyphenylene sulfide (PPS) are the two extensively used polymers in bonded PMs [15,16]. PPS is a semi-crystalline high temperature performance thermoplastic polymer with a high melting point of 275–285 °C. Nylon 12 (or nylon 6,6) is a crystalline thermoplastic polymer with a melting point of 178–180 °C. Nylon can be loaded with up to 70 vol% of magnetic particles filling [3], whereas PPS is reported to load up to 61 vol% of NdFeB micro particles [15]. Nylon bonded magnets can perform well only up to 150 °C. To achieve higher temperature thermal stability, PPS is an option that can perform well up to 180 °C and higher [15]. The polymer embedded magnetic particles surface are well coated with polymer, which increases the PM corrosion resistance as well as reduces the eddy current loss, which is very essential property that is required for electric machines [17]. 

A computer-aided-design (CAD) model based on Solidworks or Fusion 360 or Rhino is used in AM to produce complex shaped magnets with minimal or no waste followed by post-heat-treatment processing. It provides flexibility to create a wide range of shapes and designs for relatively limited quantity parts independent from the specific molding tools. Either magnetic-particles-loaded polymer is extruded through the printing nozzle, and printed layer-by-layer fashion or laser melting is applied to appropriately prepared precursor powder. The thickness of the printed layers depends on the printing nozzle diameter, scanning speed, and magnetic particle size of the magnet polymer composite materials, which altogether determine the printing resolution unit cell also known as voxel size [18,19]. The desired materials properties may require post-treatment of printed products. Some of the recent examples of AM fabricated PMs can be found in the literature [4,5,6,20].

AM can provide the near-net shaped NdFeB permanent magnet fabrication even with powder precursors while using laser melting resulting in magnets with an energy product of 5.65 MGOe comparable to typical injection molded bonded PMs [7]. It can reduce the cost that is associated with processing time, molding dies, and reduce the loss of magnetic materials during magnet production. However, the 3D-printed PMs contain defects, such as cracks and porosities, which do not meet certain requirements for industrial applications. The bonded permanent magnets are good alternative for mechanically flexible and moderately strong PMs. PPS bonded magnets are more thermally stable than nylon-bonded PMs. However, the viscosity of the PPS limits the dispersion of magnetic microparticles in it and, hence, reduces the volume fraction of them needed for high magnetic flux. Here, we report our successful AM fabrication, characterization, mechanical, and magnetic properties of PPS-bonded isotropic NdFeB PMs and compared them with nylon bonded PMs.

## 2. Experimental Methods

**Figure 1:**
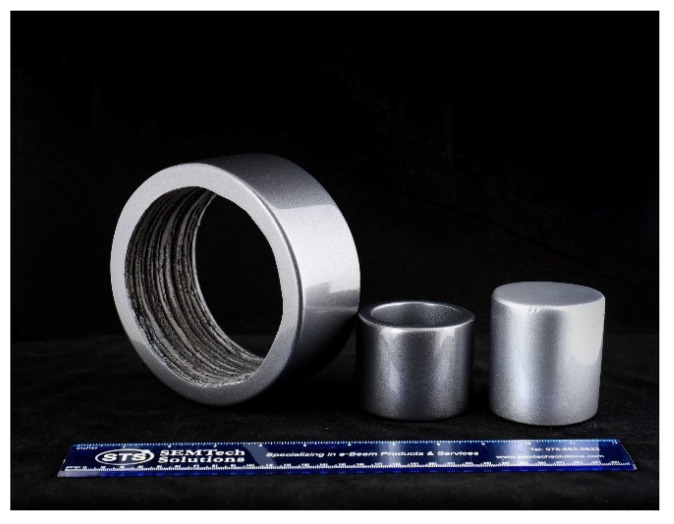
Additive Manufacturing (AM) printed NdFeB polyphenylene sulfide (PPS) hollow and solid cylinder shaped bonded PMs. Printed magnet outer surfaces were polished and coated with resin.

**Figure 2:**
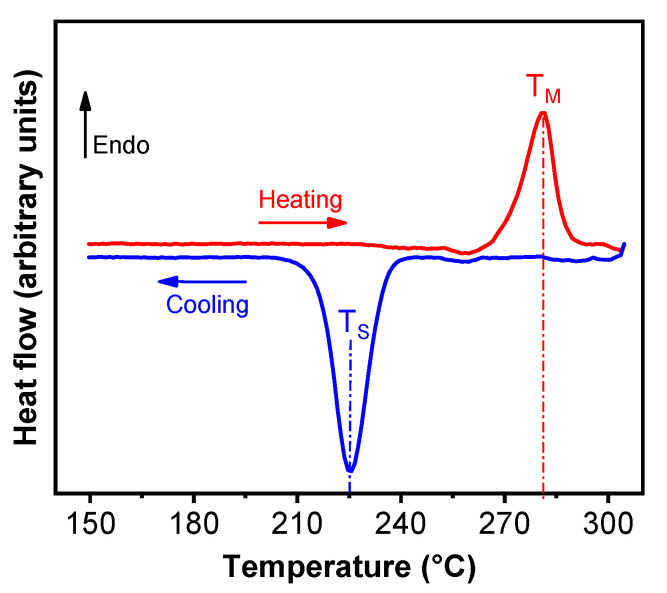
Differential scanning calorimetry (DSC) thermograms showing heat flow and thermal characteristics of NdFeB-PPS composite pellets.

Commercial PPS isotropic NdFeB composite (37:63 volume ratio) was purchased from integrated magnetics limited (Part number: L-3082) (Hong Kong). Bonded magnets were three-dimensional (3D) printed using Big Area Additive Manufacturing (BAAM) printer (Cincinnati Inc., Harrison, OH, USA) at Manufacturing Demonstration Facility in Oak Ridge National Laboratory as explained previously [3]. BAAM is specially designed as FDM based printer that can extrude polymer composite beads using a single screw extruder [21]. The geometric design and printing thickness of the depositing layers are scripted in the standard triangular language (.STL) to supply printing instruction to the BAAM printer. As obtained magnet polymer composite pellets were used as the printing material which was extruded through the Barrell inside a 5-zone furnace maintained at melt temperature range from 305, 316, 321, 321, and 321 °C with a nozzle diameter of 5.08 mm (0.2″) maintained in the range 318–324 °C. The printer bed was maintained at ~95 °C. The slightly heated printing bed helps to maintain a temperature gradient to spread the composite material to form a smooth base layer which forms the foundation for the successive iterations. The extrusion rate of 25.4 mm (1″) per sec with a layer thickness of 5.08 mm (0.2″) was maintained throughout the printing process. During printing, Argon gas was fed into the system to prevent any magnet oxidation. Figure 1 shows some examples of the printed shapes, such as hollow and solid cylinders. Differential scanning calorimetry (DSC) measurements were carried out at 10 °C/min in a Helium atmosphere on NdFeB PPS polymer composite pellet to determine its melting and solidification temperatures. The results (Figure 2) will be discussed in Section 3. The morphology of the NdFeB magnet in PPS binder was studied by scanning electron microscopy (SEM) (Zeiss Merlin, Pleasanton, CA, USA). 

Magnetic properties were determined using a vibrating sample magnetometer (Quantum Design PPMS^®^ VersaLab^™^, San Diego, CA, USA). The magnetic hysteresis loop and AC loss fraction up to 10 kHz of the printed bonded magnets were measured at 300 K. For the flux loss measurements, the AM fabricated bonded magnets were cut into rectangular shaped magnet specimens with approximate dimensions of 30 × 15 × 10 mm^3^ and were magnetized in a 5–7 T electromagnet at room temperature. Magnetized bonded magnets were coated with ~10 µm thick protective resin coatings (3M Scotch-Weld DP100 (designated as coating #1) and J-B Weld epoxy coating (designated as coating #2)) and they were studied for flux-loss at various operation temperatures. The mechanical properties of three sets of AM fabricated bonded magnets were determined at ambient conditions while using a servohydraulic testing machine (MTS Model # 810, Eden Prairie, MN, USA) equipped with hydraulically actuated grips and a clip-on extensometer with a gauge length of 25 mm. All of the tensile tests were carried out at a constant crosshead displacement rate of 5 mm/min. The test machine was also equipped with an alignment fixture to correct for lack of concentricity and/or angularity [22]. Dog-bone shaped samples (≈8 × 8 × 13.8 mm^3^) were cut to study the mechanical properties of the printed polymer composites. The variation in mechanical stress with extension is studied, which generally provides the elastic, ductile, and breaking stress for the bonded permanent magnets. 

## 3. Results and Discussion

The heat flow data from DSC help to understand thermal stability of the AM printed magnets. The DSC data of the NdFeB-PPS composite pellets show that the melting peak (T_M_) is around 280 °C as shown in Figure 2. The cooling crystallization temperature (T_S_), which is used as a rough guide for determining the solidification point to be around ~225 °C. The PPS bonded PM can perform well up to (225 °C) this solidification temperature, which is higher than nylon-bonded PMs (~150 °C). The peak positions in the heat flow curve correspond to melting behavior which exhibit the crystallinity and crystallization during cooling correspond to solidification. The smaller the difference between the melting point and solidification point, higher the solidification rates. 

**Figure 3:**
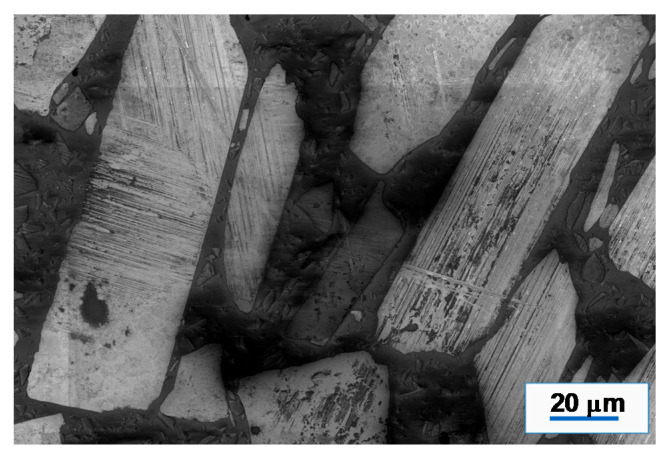
Scanning electron microscopy (SEM) micrographs of AM fabricated magnets featuring plate like morphology of NdFeB present in PPS polymer.

Similarly, the electron backscattered microstructure of the printed magnet is shown in Figure 3. The bright regions are NdFeB microstructures and the dark areas are the PPS matrix. The intermediate bright areas are either embedded NdFeB microparticles in little deeper regions or exhibiting orientation contrast. In a very careful look, the PPS polymer network includes the embedded bright and dark smaller microparticles, which are both the result of orientation contrast and polymer depth contrast. The NdFeB particles have plate-like morphology with widths that are in the range of 20–40 μm.

**Figure 4:**
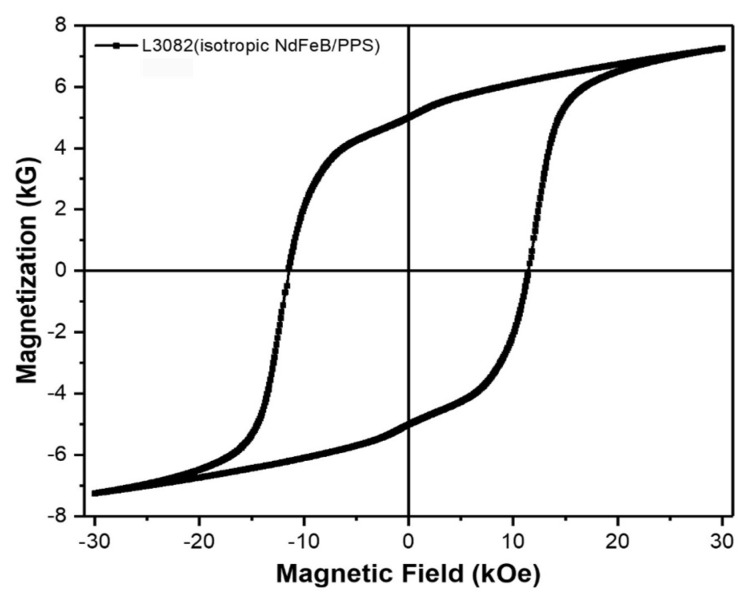
Magnetic hysteresis loop of AM fabricated NdFeB PPS bonded PM. The magnetic properties are not degraded during printing.

**Figure 5:**
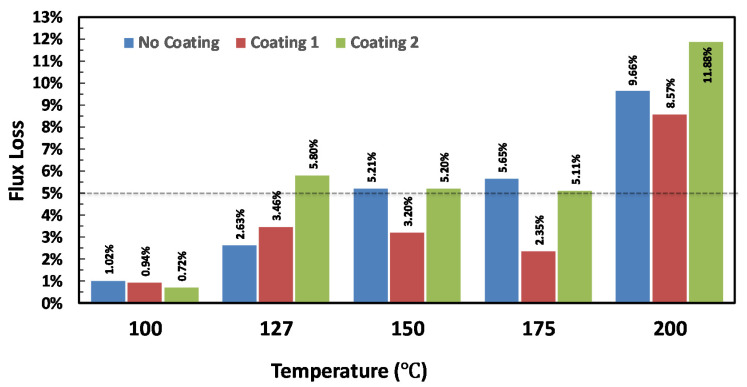
Temperature variation of the flux-loss% over 1000 h annealing at various temperatures with and without protective resin coating to mimic the permanent magnet operation cycle. Magnetized bonded magnets were coated with ~10 µm thick protective resin coatings (3M Scotch-Weld DP100 (designated as coating #1) and J-B Weld epoxy coating (designated as coating #2)).

The room temperature magnetization curve of the AM printed magnet is presented in Figure 4. The remanence and coercivity data of the printed sample almost matches with the company specifications: Br > 5 kG and H_C_ > 11 kOe and BH(max) > 5.7 MGOe, as presented in Table 1. The printing process does not change the magnetic properties noticeably from the starting pellets. A PM tends to lose its magnetization and coercivity after prolonged use at harsh operating conditions also known as aging flux-loss. A PM is considered to be thermally stable if the flux loss over 1000 h of operation does not exceed 5%. The thermal stability of magnets can be compared by measuring the room temperature flux density of the samples before and after aging at elevated temperatures (at 100, 127, 150, 175, and 200 °C for this study). The flux loss is related to a magnetization reversal mechanism occurring with rising temperature. The total flux loss is composed of recoverable losses and irreversible losses, and the latter could be due to the oxidation of the NdFeB magnet powder. The percentage of flux loss at certain temperature is a direct reflection of the thermal stability of the magnet. The flux aging loss with time for both magnets (printed; post-annealed; and, coated) are presented in Figure 5. The as printed PM loses ~5% at 150 °C. However, resin coated magnets were very stable up to 175 °C for 1000 h. The 3M Scotch-Weld DP100 resin (coating #1) coated PM showed better thermal stability over J-B Weld epoxy coating (coating #2). Resin coating was found to be very useful to improve the flux loss resistant ability and thermal stability of the bonded magnet. 

**Figure 6:**
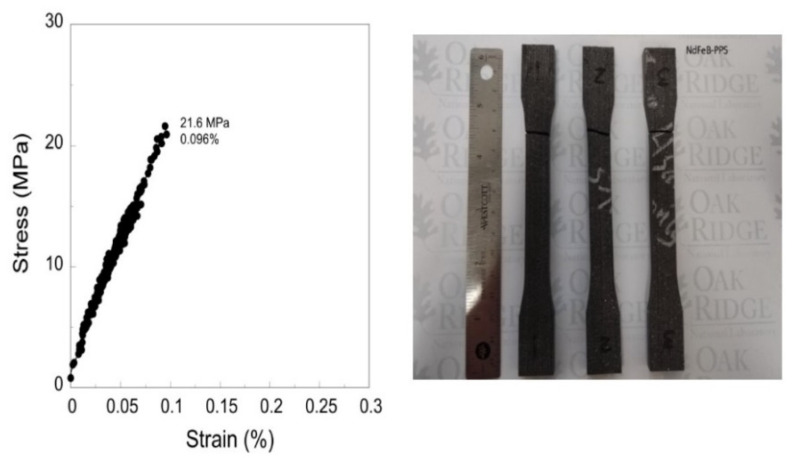
Tensile stress-displacement curve of the Big Area Additive Manufacturing (BAAM) fabricated NdFeB PPS magnets (**left**); Fractured dog-bone shaped magnets after tensile evaluation (**right**).

Figure 6 shows the tested AM fabricated NdFeB PPS bonded PMs designed for mechanical properties study. Figure 6 presents the corresponding variation of mechanical stress versus strain. The linear inclined segment up to 20 MPa tensile stress in PPS-bonded magnet along low elongation value represents its brittleness. Corresponding Young’s modulus was 21 GPa, as presented in Table 2. Being brittle thermoplastic, the PPS-bonded magnet samples break in a brittle fracture fashion beyond the 20 MPa tensile stress, as seen in Figure 6. 

The tensile stress of nylon-bonded PM is reported to be ~6 MPa [15]. The nylon-bonded magnet attains the maximum tensile strength at ~7 MPa beyond this point, the stress decreases with an increase in strain and breaks ultimately. In addition to higher thermal stability, the PPS bonded PM has a higher mechanical strength (see Table 2), almost double than AM printed nylon bonded PM [3] as well as consistent with previous injection molded bonded-NdFeB magnets [15,23]. However, it is a brittle thermoplastic and becomes less flexible than nylon-bonded PMs. The ductile region is absent in a PPS bonded PM and it ultimately breaks without much extension. 

**Figure 7:**
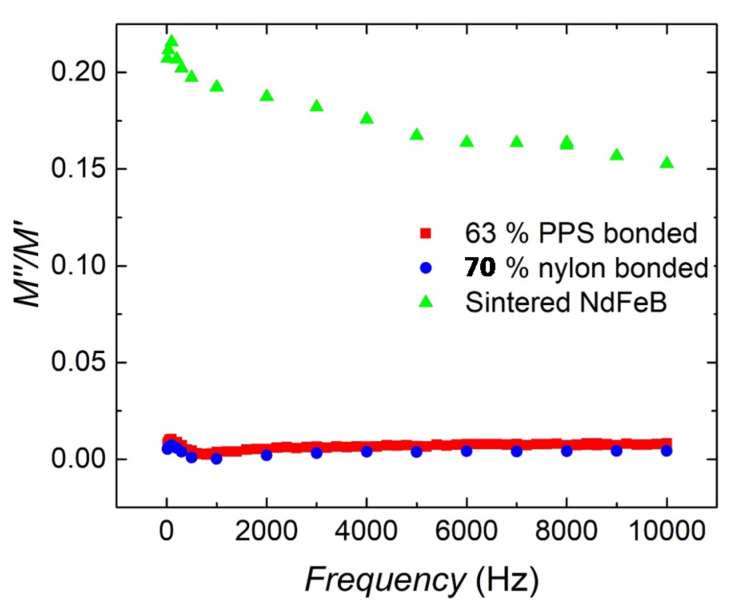
The eddy current loss fraction of 63 vol% BAAM NdFeB-PPS magnets compare with sintered NdFeB and 70 vol% BAAM NdFeB-Nylon magnets.

Recently, Li et al. [2] reported a high resistivity value of 170 mΩ.cm for 70 vol% BAAM NdFeB-nylon bonded magnets as compared to that of the state-of-the-art sintered NdFeB magnets with 150 μΩ.cm. Additionally, they reported a very low eddy current loss for 70 vol% BAAM NdFeB-nylon bonded magnets. Here, we have reported the total AC magnetization loss fraction as a function of AC magnetic field frequency (amplitude of 10 Oe) for 63 vol% BAAM NdFeB-PPS bonded magnets in Figure 7. Eddy current loss for the printed magnets is proportional to AC magnetic loss fraction $\frac{M^{\prime \prime }}{M^{\prime }}$, where $M^{\prime \prime }$ is the imaginary and $M^{\prime }$ is the real part of the magnetization. The imaginary part $M^{\prime \prime }$ represents the dissipative losses in the bonded magnets which can result in subsequent cycles of magnetization. In addition to the eddy current loss due to dissipation in electrical conductors, further losses due to irreversible domain wall motion or hysteresis loss are observed with ferromagnets. These results are also compared with anisotropic NdFeB sintered magnets (for comparison) and BAAM 70 vol% NdFeB-nylon magnets. Printed magnets were demagnetized for this measurement. The measured DC electrical resistivity, ρ, of BAAM NdFeB-PPS bonded magnets is 2.58 Ω.cm. This value is even higher than the reported value for BAAM 70 vol% NdFeB-nylon magnets. Because the eddy current losses [17,18] are proportional to 1/ρ, the printed bonded magnets will have significantly less eddy current heating. This is consistent with the eddy current loss fraction of the printed magnet which is extremely low with $\frac{M^{\prime \prime }}{M^{\prime }}$ < 1% with increasing frequency, whereas the sintered magnet exhibits a higher eddy current loss fraction of 20%. Even though the energy product of printed bonded magnets is reduced due to the incorporation of a non-magnetic PPS polymer binder, the advantages of substantial design freedom and low eddy current loss associated with high resistivity results in high conversion efficiency. These benefits will make AM magnets rival sintered magnets for certain motor applications. 

**Figure 8:**
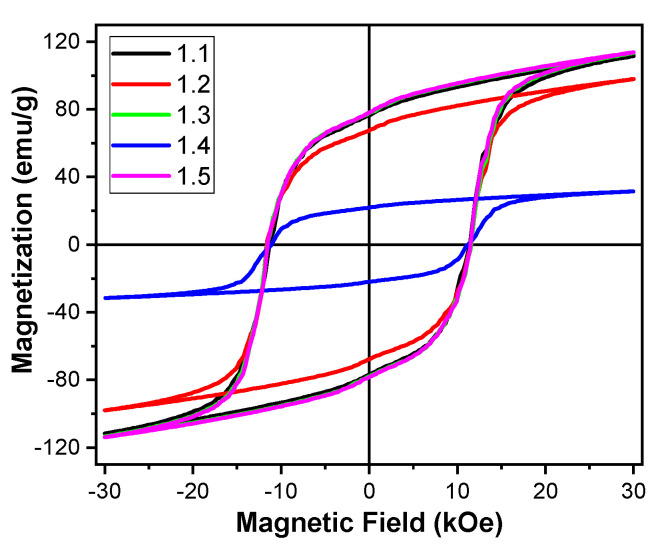
Magnetic hysteresis loop of AM fabricated NdFeB PPS bonded PM at room temperature. Sample ID: 1.1- as printed; 1.2-uncoated (dipped in pH 1.35 solution for 24 h), 1.3- coated with 3M ScotchWeld DP100 (dipped in pH 1.35 solution 24 h), 1.4- uncoated (80 °C; 95% RH; > 120 h), 1.5- coated with 3M ScotchWeld DP100 (80 °C; 95% RH; > 100 h).

The corrosion resistance of AM fabricated NdFeB PPS bonded PMs with and without the best coating of 3M identified ScotchWeld DP100 identified from the flux loss measurements (Figure 5) were tested under couple of standard industrial conditions. The magnets were soaked in a solution with a pH of 1.35 for 24 h and annealing the magnets in 95% relative humidity (RH) at 80 °C for >100 h. Figure 8 presents the room temperature magnetization curves of the AM printed magnets with and without coating under two corrosion resistance test conditions. The coercivity and saturation magnetization values of these magnets are presented in Table 3. Coated magnets survived both aggressive conditions. However, uncoated magnets drastically degraded in both test conditions. From these results, we can conclude that printed magnets may need suitable coating conditions identified in this work.

## 4. Conclusions

AM fabrication, characterization, thermal stability, mechanical, and magnetic properties of BAAM printed isotropic PPS-NdFeB bonded PMs is studied. The AM fabrication is a useful technology for the printing of complex shaped materials and tools in small scale requirements. 63:37 vol% of NdFeB:PPS bonded PM was 3D printed, heat treated, and various physical properties: mechanical, thermal stability, and magnetic flux-loss over 1000 h are studied at several operating temperatures. Industry standards require magnetic flux loss <5% after 1000 h of operation at any given temperature. AM printed, PPS bonded, and 3M Scotch-Weld DP100 coated surface coated bonded NdFeB PM meets the industrial stability criterion up to 175 °C with flux loss as low as 2.35% over 1000 h heat treatment. The tensile strength of the PPS bonded PM is almost twice (~20 MPa) that of the corresponding nylon bonded PM. The AM manufactured PPS-bonded PM shows higher thermal stability and more robust mechanical properties needed for the high-performance machine parts in comparison to nylon bonded AM magnets. This study demonstrates that AM fabricated bonded magnets with reduced manufacturing waste may potentially offset some critical rare earth element demand through targeted use in high-efficiency motors.

## Figures and Tables

**Table 1:** Magnetic properties of AM fabricated NdFeB-PPS bonded PM measured at room temperature.

Residual Magnetization (kG)	Saturation Magnetization (kG)	Coercivity (kOe)	BHmax (MGOe)	Density (g/cm^3^)
5.0	7.3	11.4	5.4	4.85

**Table 2:** Mechanical properties of AM fabricated NdFeB-PPS bonded PM measured at room temperature.

Sample #	Tensile Strength (MPa)	Tensile Strain (%)	Young’s Modulus (GPa)
1	21.60	0.096	20.00
2	18.90	0.082	22.60
3	20.60	0.091	22.00
Average	20.37	0.090	21.53
Std. Deviation	1.37	0.007	1.36

**Table 3:** Magnetic properties of corrosion resistance tested AM fabricated NdFeB-PPS bonded PM measured at room temperature. Sample ID #: 1.1- as printed; 1.2-uncoated (dipped in pH 1.35 solution for 24 h), 1.3- coated with 3M ScotchWeld DP100 (dipped in pH 1.35 solution 24 h), 1.4- uncoated (80 °C; 95% RH; >120 h), 1.5- coated with 3M ScotchWeld DP100 (80 °C; 95% RH; >100 h).

Sample ID	Coercivity (kOe)	Saturation Magnetization (emu/g)
1.1	11.33	111.5
1.2	11.62	98
1.3	11.57	113.3
1.4	11.11	32
1.5	11.53	113.7

## References

[1] Von Petersdorff-Campen K., Hauswirth Y., Carpenter J., Hagmann A., Boes S., Schmid Daners M., Penner D., Meboldt M. (2018). 3D Printing of Functional Assemblies with Integrated Polymer-Bonded Magnets Demonstrated with a Prototype of a Rotary Blood Pump. Appl. Sci..

[2] Li L., Jones K., Sales B., Pries J.L., Nlebedim I.C., Jin K., Bei H., Post B.K., Kesler M.S., Rios O. (2018). Fabrication of highly dense isotropic Nd-Fe-B nylon bonded magnets via extrusion-based additive manufacturing. Addit. Manuf..

[3] Li L., Tirado A., Nlebedim I.C., Rios O., Post B., Kunc V., Lowden R.R., Lara-Curzio E., Fredette R., Ormerod J. (2016). Big area additive manufacturing of high performance bonded NdFeB magnets. Sci. Rep..

[4] Li L., Post B., Kunc V., Elliott A.M., Parans Paranthaman M. (2017). Additive manufacturing of near-net-shape bonded magnets: Prospects and challenges. Scr. Mater..

[5] Gandha K., Li L., Nlebedim I.C., Post B.K., Kunc V., Sales B.C., Bell J., Parans Paranthaman M. (2018). Additive manufacturing of anisotropic hybrid NdFeB-SmFeN nylon composite bonded magnets. J. Magn. Magn. Mater..

[6] Gandha K., Nlebedim I.C., Kunc V., Lara-Curzio E., Fredette R., Parans Paranthaman M. (2020). Additive manufacturing of highly dense anisotropic Nd–Fe–B bonded magnets. Scr. Mater..

[7] Jaćimović J., Binda F., Herrmann L.G., Greuter F., Genta J., Calvo M., Tomse T., Simon R.A. (2017). Net Shape 3D Printed NdFeB Permanent Magnet. Adv. Eng. Mater..

[8] Tomše T., Samardžija Z., Scherf L., Kessler R., Kobe S., Rozman K.Z., Sturm S. (2020). A spark-plasma-sintering approach to the manufacture of anisotropic Nd-Fe-B permanent magnets. J. Magn. Magn. Mater..

[9] Lamarre J.M., Bernier F. (2019). Permanent Magnets Produced by Cold Spray Additive Manufacturing for Electric Engines. J. Therm. Spray. Technol..

[10] Huber C., Mitteramskogler G., Goertler M., Teliban L., Groenefeld M., Suess D. (2020). Additive Manufactured Polymer-Bonded Isotropic NdFeB Magnets by Stereolithography and Their Comparison to Fused Filament Fabricated and Selective Laser Sintered Magnets. Materials.

[11] Sridharan N., Cakmak E., List F.A., Ucar H., Constantinides S., Babu S.S., McCall S.K., Parans Paranthaman M. (2018). Rationalization of solidification mechanism of Nd–Fe–B magnets during laser directed-energy deposition. J. Mater. Sci..

[12] Löwa N., Fabert J.M., Gutkelch D., Paysen H., Kosch O., Wiekhorst F. (2019). 3D-printing of novel magnetic composites based on magnetic nanoparticles and photopolymers. J. Magn. Magn. Mater..

[13] Lee R.W., Croat J.J. (1978). Method of Making a Laminated Rare Earth Metal-Cobalt Permanent Magnet Body. U.S. Patent.

[14] Parans Paranthaman M., Shafer C.S., Elliott A.M., Siddel D.H., McGuire M.A., Springfield R.M., Martin J., Fredette R., Ormerod J. (2016). Binder Jetting: A Novel NdFeB Bonded Magnet Fabrication Process. JOM.

[15] Garrell M.G., Shih A.J., Ma B.M., Lara-Curzio E., Scattergood R.O. (2003). Mechanical properties of Nylon bonded Nd–Fe–B permanent magnets. J. Magn. Magn. Mater..

[16] Brown D., Ma B.M., Chen Z. (2002). Developments in the processing and properties of NdFeB-type permanent magnets. J. Magn. Magn. Mater..

[17] Shokrollahi H., Janghorban K. (2007). Soft magnetic composite materials (SMCs). J. Mater. Process. Technol..

[18] Bikas H., Stavropoulos P., Chryssolouris G. (2016). Additive manufacturing methods and modelling approaches: A critical review. Int. J. Adv. Manuf. Technol..

[19] Wang X., Jiang M., Zhou Z., Guo J., Hui D. (2016). 3D printing of polymer matrix composites: A review and prospective. Compos. Part B Eng..

[20] Huber C., Abert C., Bruckner F., Groenefeld M., Muthsam O., Schuschnigg S., Sirak K., Thanhoffer R., Teliban I., Vogler C. (2016). 3D print of polymer bonded rare-earth magnets, and 3D magnetic field scanning with an end-user 3D printer. Appl. Phys. Lett..

[21] Duty C.E., Kunc V., Compton B., Post B., Erdman D., Smith R., Lind R., Lloyd P., Love L. (2017). Structure and mechanical behavior of Big Area Additive Manufacturing (BAAM) materials. Rapid Prototyp. J..

[22] Garrell M.G., Shih A.J., Lara-Curzio E., Scattergood R.O. (2003). Finite-Element Analysis of Stress Concentration in ASTM D 638 Tension Specimens. J. Test. Eval..

[23] Hemrick J.G., Lara-Curzio E., Liu K., Ma B.M. (2004). Mechanical properties of thermally cycled nylon bonded Nd-Fe-B permanent magnets. J. Mater. Sci..

